# The outcome of patients with myocardial infarction with non-obstructive coronary arteries (MINOCA) and impaired kidney function: a 3-year observational study

**DOI:** 10.1007/s11255-021-02794-x

**Published:** 2021-02-18

**Authors:** Malgorzata Zalewska-Adamiec, Jolanta Malyszko, Ewelina Grodzka, Lukasz Kuzma, Slawomir Dobrzycki, Hanna Bachorzewska-Gajewska

**Affiliations:** 1 Department of Invasive Cardiology, Medical University of Bialystok, Białystok, Poland; 2 Department of Nephrology, Dialysis and Internal Medicine, Warsaw Medical University, Banacha 1 a, 02-097 Warsaw, Poland; 3 Department of Clinical Medicine, Medical University of Bialystok, Białystok, Poland

**Keywords:** Myocardial infarction, MINOCA, Chronic kidney disease, Glomerular filtration, Takotsubo, Myocarditis

## Abstract

**Background:**

Myocardial infarction with nonobstructive coronary arteries (MINOCA) constitutes about 10% of the cases of acute coronary syndromes (ACS). It is a working diagnosis and requires further diagnostics to determine the cause of ACS.

**Methods:**

In this study, 178 patients were initially diagnosed with MINOCA over a period of 3 years at the Department of Invasive Cardiology of the University Clinical Hospital in Białystok. The value of estimated glomerular filtration rate (eGFR) was calculated for all patients. The patients were divided into 2 groups depending on the value of eGFR: group 1—53 patients with impaired kidney function (eGFR < 60 mL/min/1.73 m^2^; 29.8%) and group 2—125 patients with normal kidney function (eGFR ≥ 60 mL/min/1.73 m^2^; 70.2%).

**Results:**

In group 1, the mean age of patients was significantly higher than that of group 2 patients (77.40 vs 59.27; *p* < 0.0001). Group had more women than group 2 (73.58% vs 49.60%; *p* = 0.003). Group 1 patients had higher incidence rate of arterial hypertension (92.45% vs 60.80%; *p* < 0.0001) and diabetes (32.08% vs 9.60%; *p* = 0.0002) and smoked cigarettes (22.64% vs 40.80%; *p* = 0.020). Group 1 patients had higher incidence rate of pulmonary edema, cardiogenic shock, sudden cardiac arrest (13.21% vs 4.00%; *p* = 0.025), and pneumonia (22.64% vs 6.40%; *p* = 0.001). After the 37-month observation, the mortality rate of the patients with MINOCA was 16.85%. Among group two patients, more of them became deceased during hospitalization (7.55% vs 0.80%; *p* = 0.012), followed by after 1 year (26.42% vs 7.20%; *p* = 0.0004) and after 3 years (33.96% vs 9.6%; *p* < 0.0001). Multivariate analysis revealed that the factors increasing the risk of death in MINOCA are as follows: older age, low eGFR, higher creatinine concentration, low left ventricular ejection fraction, and ST elevation in ECG.

**Conclusion:**

Impaired kidney function is diagnosed in every third patient with MINOCA. Early and late prognosis of patents with MINOCA and renal dysfunction is poor, and their 3-year mortality is comparable to patients with myocardial infarction with significant stenosis of the coronary arteries and impaired kidney function.

## Introduction

Myocardial infarction with nonobstructive coronary arteries (MINOCA) is a condition that constitutes about 10% of the cases of acute coronary syndrome (ACS). It is diagnosed in patients with clinical symptoms of acute myocardial infarction in whom cardiac catheterization does not reveal major epicardial arterial stenosis (> 50%). MINOCA is a working diagnosis and it requires patient-specific diagnosis search for the underlying cause for this acute cardiac status [1–3].

There are potentially multiple mechanisms that lead to MINOCA. The most common cause can be the rupture or ulceration of atherosclerotic plaque, which is typical of type 1 myocardial infarction, as well as by the dissection of the coronary artery, both subject to the atherosclerotic lesion and not. It can also be caused by the disturbances in the coronary microcirculation, coronary thrombo-embolism, or coronary artery spasm, which is typical of type 2 myocardial infarction. Other causes for MINOCA include myocardial damage such as the Takotsubo syndrome (TTS) and myocardial inflammation [1, 4].

Chronic kidney disease (CKD) increases the risk of cardiovascular diseases by several-fold. Apart from the elevated risk of cardiovascular diseases, renal dysfunction in patients with cardiological problems is associated with more advanced disease and its more severe course, higher risk of complications, longer hospitalization period, and poorer short- and long-term prognosis [5–8].

The aim of the study was to assess the incidence of impaired kidney function in patients with MINOCA and to evaluate the impact of renal impairment on the early and long-term prognosis of these patients.

## Methods

### Study population

A total of 1640 patients were hospitalized for myocardial infarction at the Invasive Cardiology Clinic of the University Clinical Hospital in Białystok over a period of 3 years (01/04/2015–30/04/2018). On admission, 178 patients (10.8%) were diagnosed with MINOCA.

MINOCA was initially diagnosed based on the criteria met for the acute myocardial infarction contained in the fourth universal definition of myocardial infarction [9] and absence of significant atherosclerotic changes in coronary arteries observed in the coronarography. In the case of patients, whose laboratory and imaging tests did not show clear cause for ACS (i.e. TTS, myocardial inflammation, and type 2 myocardial inflammation), MINOCA remained the final diagnosis. The Takotsubo syndrome was diagnosed based on the criteria proposed by Mayo Clinic [10] and based on the criteria included in the statement of the European Society of Cardiology [11]. Myocardial inflammation was diagnosed based on the definition of the ESC 2013 Myocarditis Task Force [12]. Type 2 myocardial infarction was diagnosed if rhythm abnormality, increased arterial blood pressure, coronary microvascular dysfunction, or vasospastic angina were considered to be the cause of the myocardial infarction.

All 178 patients with the initial diagnosis of MINOCA were included in the study. In the whole group, eGFR was estimated based on the creatinine concentration measured on admission to the clinic. The value of the estimated glomerular filtration rate (eGFR) was calculated by using the Chronic Kidney Disease Epidemiology Collaboration formula (CKD-EPI) [13]. eGFR < 60 mL/min/1.73 m^2^ was present in 44 patients, eGFR < 30 mL/min/1.73 m^2^ was estimated in 8 patients and eGFR < 15 mL/min/1.73 m^2^ was observed in 1 patient.

The patients were divided into two groups depending on the values of eGFR: group 1—patients with eGFR < 60 mL/min/1.73 m^2^ and group 2—patients with eGFR ≥ 60 mL/min/1.73 m^2^.

This study was approved by the Bioethics Committee of the Medical University of Bialystok.

### Coronary angiography

Classic coronary catheterization was performed on 174 patients, whereas coronary artery computed tomographic (CT) scan was performed on 4 juvenile patients. The severity of the coronary disease was determined based on a visual examination of atherosclerotic lesions performed by a cardiologist at the time of the test:

- Normal coronary arteries—arteries without atherosclerotic lesions
- Minor atherosclerotic lesions—atherosclerotic lesions < 30%
- Major atherosclerotic lesions—lesions ≥ 30%, but < 50%.

### Follow-up examination

The 3-year observational study was conducted based on data on deaths of the Division of Data Sharing of the Ministry of Digitalization (Department of National Systems). Mortality due to any cause was assumed as the main end-point of the analysis.

### Statistical analysis

The obtained data were statistically analyzed. To compare the quantitative data, we used Student’s *t* test and Mann–Whitney *U* test, whereas to compare the qualitative data, we performed a Chi-square test and Fisher test. We conducted survival analysis with the Kaplan–Meier method and compared the groups by the log-rank test. The univariate and multivariate analysis was performed with logistic regression method. The analysis, apart from the factors in which the tested groups differed significantly, also included the factors of potential significance for the prognosis of patients with ACS (age, female gender, hypertension, hypercholesterolaemia, diabetes, ST-segment elevation, BMI < 20, LVEF < 40%, eGFR, hemoglobin, leukocytes, troponin, LDL cholesterol, BNP, HbA1c and creatinine). The multivariate logistic regression was performed using the backward stepwise Wald method. Additionally, a multi-factor logistic regression excluding age was performed. *p* value of < 0.05 was assumed as statistically significant. The statistical analysis was performed using STATISTICA 13.1 software.

## Results

### Studied population and group comparison

**Fig. 1:**
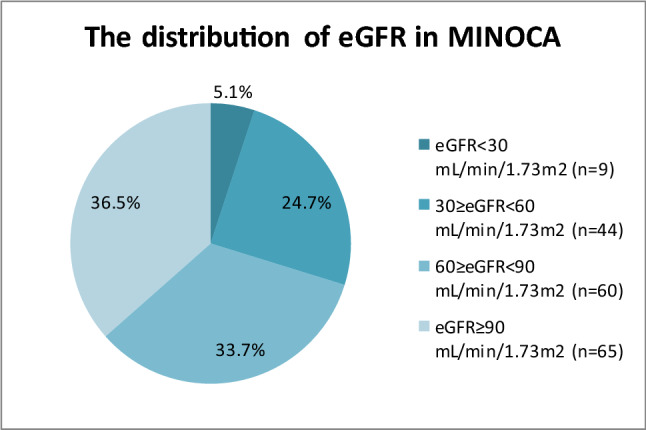
The distribution of eGFR (mL/min/1.73 m^2^) in MINOCA (*N* = 178)

Out of 178 patients with MINOCA, 53 patients were diagnosed with reduced renal function (their eGFR value was < 60 mL/min/1.73 m^2^; 29.8%), whereas the remaining 125 patients (70.2%) showed normal glomerular filtration rate (their eGFR value was ≥ 60 mL/min/1.73 m^2^). Figure 1 presents detailed eGFR distribution throughout the study group.

The mean age of patients with MINOCA was 64.67 years. The mean age of group 1 was significantly higher than that in group 2. Women comprised 56.74% of the studied group, and the number of women in group 1 was considerably higher than that of group 2 (73.58% vs 49.60%). Patients in group 1 had a higher incidence of arterial hypertension and diabetes than that of patients in group 2, and they smoked cigarettes significantly less frequently compared to group 2 patients (Table 1).

**Table 1:** Clinical and demographic characteristics—comparison of the groups of patients with MINOCA (*N* = 178)

	MINOCA *N* = 178 (SD) *N* (%)	Group 1 eGFR < 60 mL/min/1.73 m^2^ *N* = 53 (SD) *N* (%)	Group 2 eGFR ≥ 60 mL/min/1.73 m^2^ *N* = 125 (SD) *N* (%)	*p*
Age (years)	64.67 (16.31)	77.40 (10.52)	59.27 (15.39)	** < 0.0001**
Female sex (%)	101 56.74%	39 73.58%	62 49.60%	**0.003**
Body mass index (BMI) kg/m^2^	27.29 (5.06)	26.62 (4.21)	27.58 (5.84)	0.297
History of malignancy (%)	18 10.11%	7 13.21%	11 8.80%	0.372
History of hypertension (%)	125 70.22%	49 92.45%	76 60.80%	** < 0.0001**
Hyperlipidemia (%)	106 59.55%	31 58.49%	75 60.00%	0.851
Smoking (%)	63 35.39%	12 22.64%	51 40.80%	**0.020**
Family history of coronary artery disease (%)	31 17.42%	6 11.32%	25 20.00%	0.162
Diabetes mellitus (%)	29 16.29%	17 32.08%	12 9.60%	**0.0002**
Anxiety/depression (%)	7 3.93%	1 1.89%	6 4.80%	0.361
Thyroid disorders (%)	29 16.29%	8 15.09%	21 16.80%	0.777
Asthma, COPD (%)	21 11.80%	8 15.09%	13 10.40%	0.375
Previous MI (%)	29 16.29%	10 18.87%	19 15.20%	0.544

Significant *p* values are indicated in bold

*COPD* chronic obstructive pulmonary disease, *MI* myocardial infarction

Group 1 was found to have significantly lower mean left ventricular ejection fraction. Group 2 patients more often had normal coronary arteries in angiography, whereas group 1 patients showed a greater frequency of minor atherosclerotic lesions in coronary arteries. Electrocardiograms revealed a significantly longer QTc interval among group 1 patients at the admission to the clinic and after several days of hospitalization than that group 2 patients (Table 2).

**Table 2:** Diagnostic tests (echocardiography, coronarography, ECG)—comparison of the groups of patients with MINOCA (*N* = 178)

	MINOCA *N* = 178 (SD) *N* (%)	Group 1 eGFR < 60 mL/min/1.73 m^2^ *N* = 53 (SD) *N* (%)	Group 2 eGFR ≥ 60 mL/min/1.73 m^2^ *N* = 125 (SD) *N* (%)	*p*
LVEF (%)	46.19 (12.42)	42.84 (13.95)	47.54 (11.60)	**0.023**
No atherosclerotic changes in coronary arteries (%)	45 25.28%	5 9.43%	40 32.00%	**0.001**
Insignificant stenoses < 50% (%)	133 74.72%	48 90.57%	85 68.00%	**0.0015**
Insignificant stenoses < 30% (%)	35 19.66%	7 13.21%	28 22.40%	0.158
Insignificant stenoses ≥ 30% but < 50% (%)	98 55.06%	41 77.36%	57 45.60%	** < 0.0001**
ECG–ST-segment elevation (%)	70 39.33%	21 39.62%	49 39.20%	0.958
ECG–ST-segment depression (%)	26 14.61%	8 15.09%	18 14.40%	0.905
Negative T waves (%)	72 40.45%	24 45.28%	48 38.40%	0.392
QTc on admission (ms)	459.61 (36.51)	474.21 (41.99)	453.36 (32.30)	**0.0008**
QTc after a few days (ms)	458.90 (40.26)	476.85 (40.87)	451.15 (37.82)	**0.0002**

Significant *p* values are indicated in bold

*ECG* electrocardiogram, *LVEF* left ventricular ejection fraction

Laboratory studies revealed significantly higher glycemia and higher values of HbA1c and B-type natriuretic peptide (BNP) in group 1 patients. Furthermore, group 1 patients had lower values of total cholesterol, LDL, and HDL fractions than that of group 2 patients (Table 3).

**Table 3:** Laboratory parameters—comparison of the groups of patients with MINOCA (*N* = 178)

	MINOCA *N* = 178 (SD) *N* (%)	Group 1 eGFR < 60 mL/min/1.73 m^2^ *N* = 53 (SD) *N* (%)	Group 2 eGFR ≥ 60 mL/min/1.73 m^2^ *N* = 125 (SD) *N* (%)	*p*
Hemoglobin (mg/dl)	13.63 (1.74)	13.29 (1.88)	13.77 (1.68)	0.090
Erytrocytes(× 10^6^/μl)	4.49 (0.59)	4.48 (0.63)	4.50 (0.58)	0.835
Hematocrit (%)	40.13 (4.65)	39.72 (5.36)	40.31 (4.35)	0.446
Leukocytes (× 10^3^/μl)	9.48 (4.36)	10.28 (5.03)	9.15 (4.04)	0.113
Thrombocytes (	246.20 (98.40)	235.70 (108.98)	250.67 (94.09)	0.355
Glucose on admission (mg/dl)	119.80 (43.33)	137.50 (60.71)	112.33 (31.15)	**0.0003**
HbA1C (%)	5.93 (0.86)	6.28 (1.16)	5.78 (0.65)	**0.0007**
Creatinine (mg/dl)	0.99 (0.42)	1.40 (0.56)	0.82 (0.15)	–
eGFR MDRD (m/min/1.72 m^2^)	73.84 (25.61)	43.04 (11.29)	86.89 (17.63)	–
eGFR CKD-EPI (m/min/1.72 m^2^)	79.27 (30.66)	42.83 (11.83)	94.72 (21.96)	–
CK (IU/L)	373.50 (670.42)	288.11 (412.69)	409.76 (754.54)	0.271
CKMB (IU/L)	75.66 (78.59)	59.98 (39.56)	82.42 (84.78)	0.067
Troponin (significant increase) (%)	100%	100%	100%	–
Troponin–mean concentration (ng/mL)	5829.70 (8555.71)	4977.59 (7448.31)	6184.12 (9013.26)	0.396
Total cholesterol (mg/dl)	178.10 (44.40)	162.48 (39.73)	184.70 (44.96)	**0.0023**
LDL (mg/dl)	108.20 (39.36)	94.08 (36.54)	114.20 (39.30)	**0.0019**
HDL (mg/dl)	48.58 (15.85)	44.04 (13.80)	50.53 (16.39)	**0.0133**
Triglycerides (mg/dl)	114.10 (69.79)	128.71 (78.12)	107.88 (65.58)	0.072
CRP (mg/l)	51.38 (85.39)	58.92 (80.16)	48.68 (87.89)	0.552
Fibrinogen (mg/l)	431.16 (142.16)	451.30 (151.66)	422.55 (138.23)	0.220
BNP (pg/mL)	597.03 (801.22)	1091.36 (1142.30)	394.80 (493.96)	** < 0.0001**
Hospitalization time (days)	4.31 (3.11)	4.02 (2.31)	4.43 (3.40)	0.419

Significant *p* values are indicated in bold

*BNP* peptyd natriuretyczny typu B, *eGFR* estimated glomerular filtration rate, *CK* creatine kinase, *LDL* low-density lipoprotein, *HDL* high-density lipoprotein, *CRP* c-reactive protein

In group 1 patients, we recorded a higher heart rate both at admission and at the end of the study. Severe complications were recorded more frequently in the acute stages of the disease, e.g. lung edema, cardiogenic shock, and sudden cardiac arrest, and during hospitalization, these patients suffered from pneumonia more frequently (Table 4). Tables 1,2,3,4and 5 list more about the remaining clinical data, laboratory testing, and pharmacotherapy.

**Table 4:** Clinical course and complications—comparison of the groups of patients with MINOCA (*N* = 178)

	MINOCA *N* = 178 (SD) *N* (%)	Group 1 eGFR < 60 mL/min/1.73 m^2^ *N* = 53 (SD) *N* (%)	Group 2 eGFR ≥ 60 mL/min/1.73 m^2^ *N* = 125 (SD) *N* (%)	*p*
RRs on admission	134.88 (25.11)	135.94 (25.31)	134.43 (25.22)	0.715
RRr on admission	77.42 (13.57)	75.64 (14.34)	78.17 (13.27)	0.258
HR on admission	80.04 (19.58)	85.58 (24.56)	77.69 (16.69)	**0.013**
HR after a few days	70.01 (15.35)	73.81 (18.70)	68.40 (13.53)	**0.041**
Retrosternal chest pain (%)	161 90.45%	46 86.79%	115 92.00%	0.631
Dyspnea (%)	13 7.30%	4 7.55%	9 7.20%	0.934
Cardiac arrest, pulmonary oedema, cardiogenic shock (%)	12 6.74%	7 13.21%	5 4.00%	**0.025**
Pneumonia (%)	20 11.24%	12 22.64%	8 6.40%	**0.001**
Urinary tract infection (%)	9 5.06%	4 7.55%	5 4.00%	0.323
Rhythm disturbances (%)	26 14.67%	10 18.87%	16 12.80%	0.294
Hospitalization time (days)	4.31 (3.11)	4.02 (2.31)	4.43 (3.40)	0.419

Significant *p* values are indicated in bold

*RR* blood pressure, *HR* heart rate

**Table 5:** Pharmacological treatment at discharge—comparison of the groups of patients with MINOCA (*N* = 178)

	MINOCA *N* = 178 (SD) *N* (%)	Group 1 eGFR < 60 mL/min/1.73 m^2^ *N* = 53 (SD) *N* (%)	Group 2 eGFR ≥ 60 mL/min/1.73 m^2^ *N* = 125 (SD) *N* (%)	*p*
Clopidogrel	108 60.67%	36 67.92%	72 57.60%	0.197
Aspirin	158 88.76%	49 92.45%	109 87.20%	0.310
ACEI/ARB	143 80.34%	44 83.02%	99 79.20%	0.557
β-blockers	151 84.83%	43 81.13%	108 86.40%	0.370
Statin	139 78.09%	42 79.25%	97 77.60%	0.807

*ACEI* angiotensyn converting enzyme inhibitor, *ARB* angiotensin II receptor blocker

### Initial diagnosis of MINOCA and final diagnosis

After conducting additional testing, 39% of the patients with an initial diagnosis of MINOCA were further diagnosed with TTS, and MINOCA remained the final diagnosis for the same percentage of patients. Type 2 myocardial infarction was diagnosed in 15 patients; in 4 patients with vasospastic angina, in 5 patients due to an increase in blood pressure, in 2 patients with arrhythmias, and in 4 patients due to coronary microcirculation disorders.

**Fig. 2:**
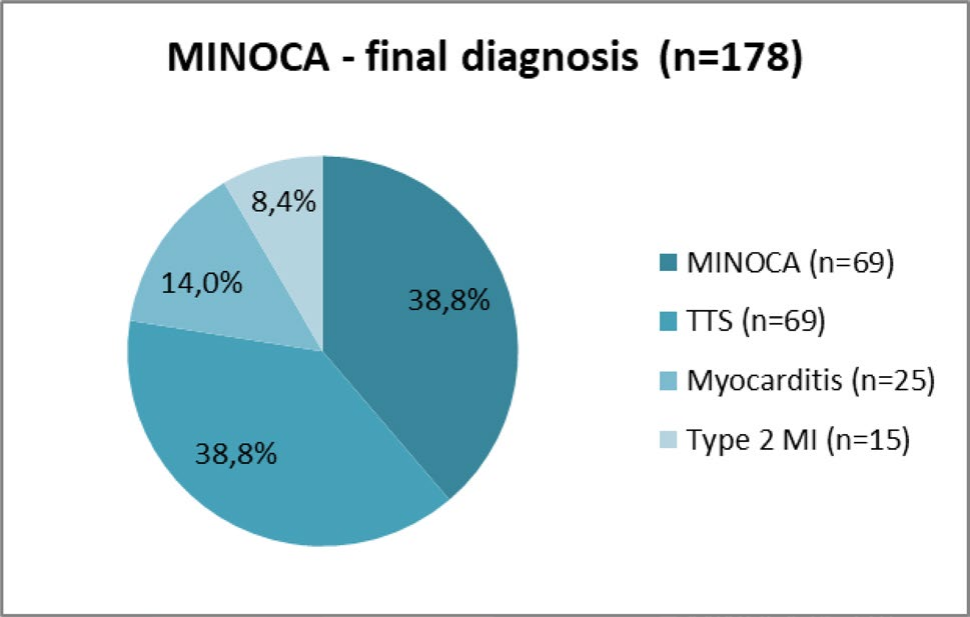
The distribution of final diagnosis in total MINOCA group (*N* = 178)

**Fig. 3:**
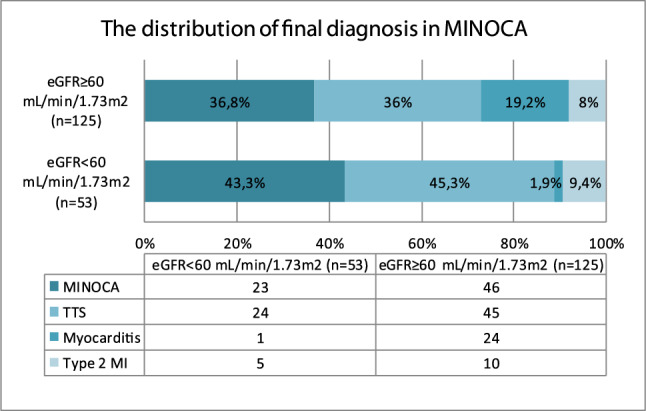
The distribution of final diagnosis in 2 MINOCA groups (*N* = 178)

According to our results, group 2 patients had a higher number of cases of myocardial inflammation than that of group 1 patients (19.2% vs 1.9%; *p* = 0.002). Figures 2 and 3 show the remaining figures and percentages for the distribution of final diagnoses in MINOCA groups.

### The mortality during the 3-year observation

**Fig. 4:**
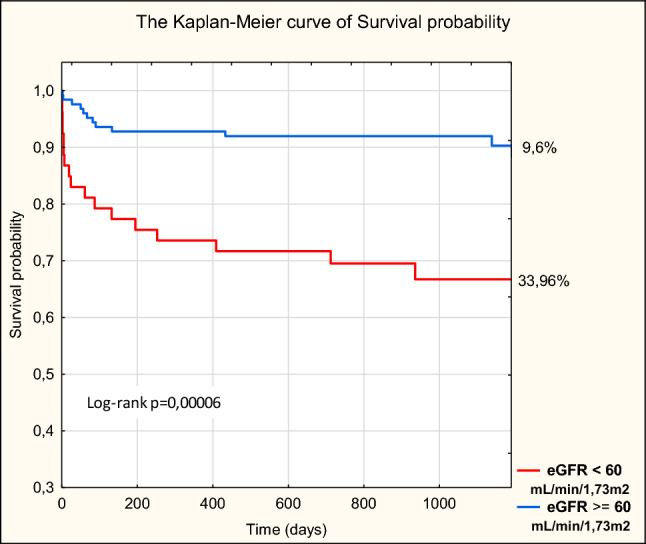
The Kaplan–Meier curve of survival probability of patients with MINOCA (*N* = 178)

The mean follow-up period was 37.16 months (3 years and 1 month). During the study period, 30 patients (16.85%) died. Group 1 patients had a significantly higher mortality rate than that of group 2 patients based on 1-month, 6-month, 1-year, and 3-year follow-up analysis (Table 6, Fig. 4).

**Table 6:** Comparison of mortality rate between the two MINOCA groups (*N* = 178)

	MINOCA *N* = 178 (SD) *N* (%)	Group 1 eGFR < 60 mL/min/1.73 m^2^ *N* = 53 (SD) *N* (%)	Group 2 eGFR ≥ 60 mL/min/1.73 m^2^ *N* = 125 (SD) *N* (%)	*p*
In hospital (%)	5 2.81%	4 7.55%	1 0.80%	**0.012**
1 months (%)	12 6.74%	9 16.98%	3 2.40%	**0.0003**
6 months (%)	21 11.80%	12 22.64%	9 7.20%	**0.003**
1 year (%)	23 12.92%	14 26.42%	9 7.20%	**0.0004**
3 year (%)	30 16.85%	18 33.96%	12 9.6%	** < 0.0001**

Significant *p* values are indicated in bold

Analysis of a 3-year mortality rate depending on the final diagnosis revealed a significantly higher mortality rate among patients with TTS and renal dysfunction (Table 7).

**Table 7:** Distribution of deaths in both MINOCA groups depending on the final diagnosis (*N* = 178)

	MINOCA *N* = 178			Group 1 eGFR < 60 mL/min/1.73 m^2^ *N* = 53			Group 2 eGFR ≥ 60 mL/min/1.73 m^2^ *N* = 125			*p*
	*n*	Deaths (*n* = 30)		*n*	Deaths (*n* = 18)		*n*	Deaths (*n* = 12)		
		*n*	%		*n*	%		*n*	%	
MINOCA	69	12	17.4	23	6	26.1	46	6	13.0	0.175
Takotsubo	69	15	21.7	24	10	41.7	45	5	11.1	**0.003**
Myocarditis	25	1	4.0	1	0	0.0	24	1	4.2	–
Type 2 MI	15	2	13.3	5	2	40.0	10	0	0.0	–

Significant *p* value is indicated in bold

Multivariate logistic regression analysis showed that factors increasing the risk of death in MINOCA are as follows: older age, low eGFR value, higher creatinine levels, low left ventricular ejection fraction, and ST elevation in ECG record. Table 8 presents detailed data from univariate and multivariate analysis. To limit the influence of age, an additional multivariate analysis excluding age was performed. In this analysis, the factors that increase the risk of death in patients with MINOCA are ST-segment elevations (95% Cl 1.128–9.390, *p* = 0.029), LVEF < 40% (95% Cl 0.901–0.978, *p* = 0.003), hemoglobin < 12 mg% (95% Cl 0.476–0.899, *p* = 0.009) and higher creatinine level (95% Cl 1.412–28.009, *p* = 0.016).

**Table 8:** Univariate and multivariate regression analysis of risk factors of death in patients with MINOCA (*N* = 178)

Predictor	Univariable			Multivariable		
	Odd ratio	95% Cl	*p*	Odds ratio	95% Cl	*p*
Female sex	0.614	0.279–1.350	0.2245			
Age	1.049	1.017–1.082	0.0028	**1.159**	**1.064–1.264**	**0.001**
BMI < 20	0.949	0.870**–**1.035	0.2365			
eGFR CKD-EPI	0.983	0.969**–**0.997	0.0145	**1.110**	**1.047–1.177**	**0.000**
Hypertension	1.861	0.713**–**4.858	0.2042			
Hypercholesterolemia	0.383	0.171**–**0.858	0.0192			
Diabetes mellitus	1.822	0.695**–**4.776	0.2222			
ST elevation	3.539	1.531**–**8.181	0.0031	**3.294**	**1.043–10.403**	**0.042**
LVEF < 40%	0.929	0.898**–**0.961	0.0000	**0.949**	**0.909–0.991**	**0.018**
Hemoglobin	0.655	0.512**–**0.838	0.0007			
LEU	1.130	1.043**–**1.224	0.0027			
Troponin	1.000	1.000**–**1.000	0.0117			
LDL	0.980	0.968**–**0.993	0.0018			
BNP	1.001	1.000**–**1.001	0.0044			
HbA1c	1.453	0.957**–**2.206	0.0797			
Creatinin	8.162	2.606**–**25.558	0.0003	**1085.19**	**23.409–50306.55**	**0.000**

Significant *p* values are indicated in bold

*Cl* confidence interval

## Discussion

Recent studies indicate an increased interest in the cardiological scientific community of myocardial infarction with nonobstructive coronary arteries (MINOCA). Patients with the initial diagnosis of MINOCA constitute from a few to several percent of patients with myocardial infarction and, due to the unclear cause of acute coronary syndrome; therefore, they constitute a difficult patient group in terms of diagnosis and therapy. MINOCA requires numerous additional laboratory and imaging tests, and despite this, often the etiology remains unresolved and the implementation of optimal treatment impossible [1–4].

Chronic kidney disease is a well-known cardiovascular risk factor. It increases the incidence rate of cardiovascular diseases and deteriorates prognosis for these patients, as shown in numerous publications [14]. However, to the best of our knowledge, no study has focused on renal dysfunction in patients with MINOCA.

Patients with impaired renal function constitute 30–40% of the patients with acute coronary syndrome. In the Polish Registry of Acute Coronary Syndrome, PL-ACS, patients with CKD constitute 36.2% of the patients with non-ST-segment elevation myocardial infarction (NSTEMI) and 26% of the patients with ST-segment elevation myocardial infarction (STEMI) [15]. Furthermore, Margolis et al. [16] determined renal dysfunction with eGFR < 60 mL/min/1.73 m^2^ in 26% of the patients with STEMI. In our study, 1/3 of the study group were patients with impaired kidney function. Similar results with mean eGFR in MINOCA were reported by Hjort et al. [17]. However, Rakowski et al. [18] who analyzed the ORPKI registry (Polish National Percutaneous Coronary Interventions Registry), found CKD at a few percent of the patients with MINOCA and the patients with myocardial infarction with obstructive coronary arteries. These significant differences between studies are due to different criteria for diagnosing chronic kidney disease. In the presented study, before admission to the hospital, CKD was diagnosed in 20 patients. After the eGFR was assessed, renal impairment was diagnosed in a much larger group of patients. That is why it is so important to assess glomerular filtration in all patients with myocardial infarction to diagnose CKD as early as possible.

In the presented study, patients with impaired kidney function more often suffered from hypertension and diabetes. In addition, laboratory tests showed higher levels of glycemia and glycosylated hemoglobin in them; therefore, it is very likely that diabetes and hypertension are the cause of kidney function damage in these patients.

A considerable proportion of the final diagnoses is represented by Takotsubo syndrome in our studied group of patients with MINOCA. This element is highly variable in other reports, Santos et al. [19] reported it to be 8%, whereas Hausvater et al. [20] reported it to be over a dozen percent. Pais et al. [21] reported that patients with TTS constitute 27.4% of the patients with an initial diagnosis of MINOCA and Gaibazzi et al. [22] reported a similar percentage. Other studies, mainly based on registers of myocardial infarctions, have not included patients with TTS, because at the time of diagnosis of TTS, they were excluded from the group of patients with myocardial infarction.

In this study, the mortality of patients with renal dysfunction was significantly higher than that of patients with normal kidney function both during early as well as during follow-up study. After 30 days and 37 months, mortality was significantly higher in the group of patients with MINOCA and impaired renal function. Similarly, high mortalities were determined among patients with chronic kidney disease in the PL-ACS registry; the 30-day mortality rate was 11% in NSTEMI and 18.7% in the STEMI group, whereas after a 3-year observation, 38.9% of the patients in NSTEMI and 39.4% patients in STEMI group died [15]. However, Margolis et al., after an average of 38-month follow-up, determined 17.6% mortality in the group with STEMI and CKD and only 2.7% among patients with normal renal function [16].

In this study, similar to the study of Margolis et al. [16], multivariate analysis demonstrated that renal dysfunction constitutes a risk factor for patients with myocardial infarction.

In this study, depending on the final diagnosis, we obtained a significantly higher mortality rate for the patients with a final diagnosis of Takotsubo syndrome and impaired renal function than that of patients with TTS and normal kidney function. This result coincides with the previous study published by the authors. Vago et al. [23] also reported a high mortality rate in their 4-year follow-up study of on patients with MINOCA and TTS. They did not include the impact of renal dysfunction on the prognosis, but the highest percentage of patients with CKD was observed in the TTS group (36%).

In this study, significant differences in the advancement of coronary artery disease were found in patients with renal dysfunction and with normal renal function. Only a few patients with eGFR < 60 mL/min/1.73 m^2^ had normal coronary arteries, while the remaining majority had atherosclerotic lesions. Moreover, patients with CKD had more advanced coronary artery disease. Disorders of the calcium-phosphate metabolism, endothelial dysfunction and continuous oxidative stress in CKD patients not only accelerate the development of atherosclerosis, but also contribute to the rupture of unstable irrelevant atherosclerotic lesions, causing acute coronary syndrome and worsening the prognosis of patients.

Patients with myocardial infarction with nonobstructive coronary arteries are a difficult group, and patients with MINOCA and impaired kidney function constitute the most challenging group. These patients have a markedly poorer prognosis at the outset, thus requiring special care and diagnostics to determine the cause of MINOCA and implementing optimal treatment that improves the prognosis of patients.

## Conclusion

Impaired kidney function is diagnosed in every third patient with MINOCA. Early and late prognosis of patients with MINOCA and renal dysfunction is poor, and their 3-year mortality is comparable to patients with myocardial infarction with significant stenosis of the coronary arteries and impaired kidney function. Patients with MINOCA and CKD are a difficult group of patients, requiring further clinical trials with a long follow-up period.

## Study limitation

This study is of a retrospective nature. However, the greatest limitation to this study is the fact that it is a single-center study, and thus the number of patients with MINOCA included in the study is low. The follow-up study was conducted on the PESEL database, from which only information on the date of death of the patients was obtained, without details on the causes of deaths.

### What is new?

This is the first study assessing the impact of impaired kidney function on the clinical course and prognosis of patients with myocardial infarction with nonobstructive coronary arteries (MINOCA).

### What are the clinical implications?

This study revealed a markedly poorer prognosis for patients with MINOCA and renal dysfunction in early and follow-up period. Patients with MINOCA and impaired kidney function require special attention and diagnostics to determine the cause for MINOCA and implementation of efficient treatment improving the prognosis.
